# Supplementary figures and images for: Discovery of a Natural Microsporidian Pathogen with a Broad Tissue Tropism in Caenorhabditis elegans
Source: PLoS Pathog. 2016 Jun 30;12(6):e1005724. doi: 10.1371/journal.ppat.1005724 (PMC4928854; doi:10.1371/journal.ppat.1005724)

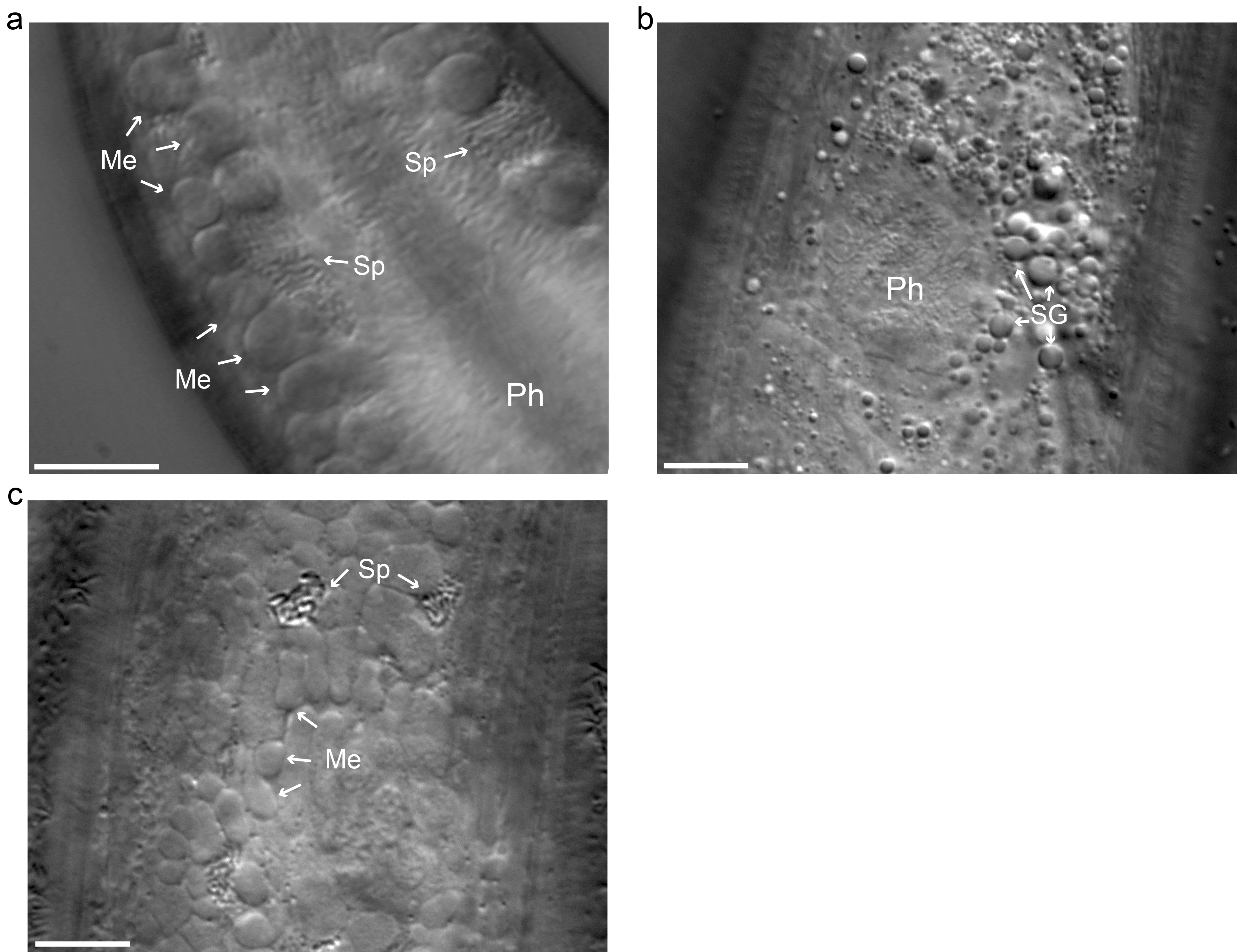

Supplement: S1 Fig — (a) The head region of a wild-caught C. elegans animal with structures resembling microsporidia at different stages of infection, including meronts (Me) and groups of spores (Sp). The pharynx (Ph) is indicated for orientation. (b) The head region of a live, uninfected N2 C. elegans for comparison, with storage granules (SG) indicated. (c) Infected mid-body region of a live animal from strain JU2807 (progeny of the animal shown in Panel a) showing both meront-like structures and groups of spores. Scale bars are 10 μm. (TIF) [file ppat.1005724.s001.tif]

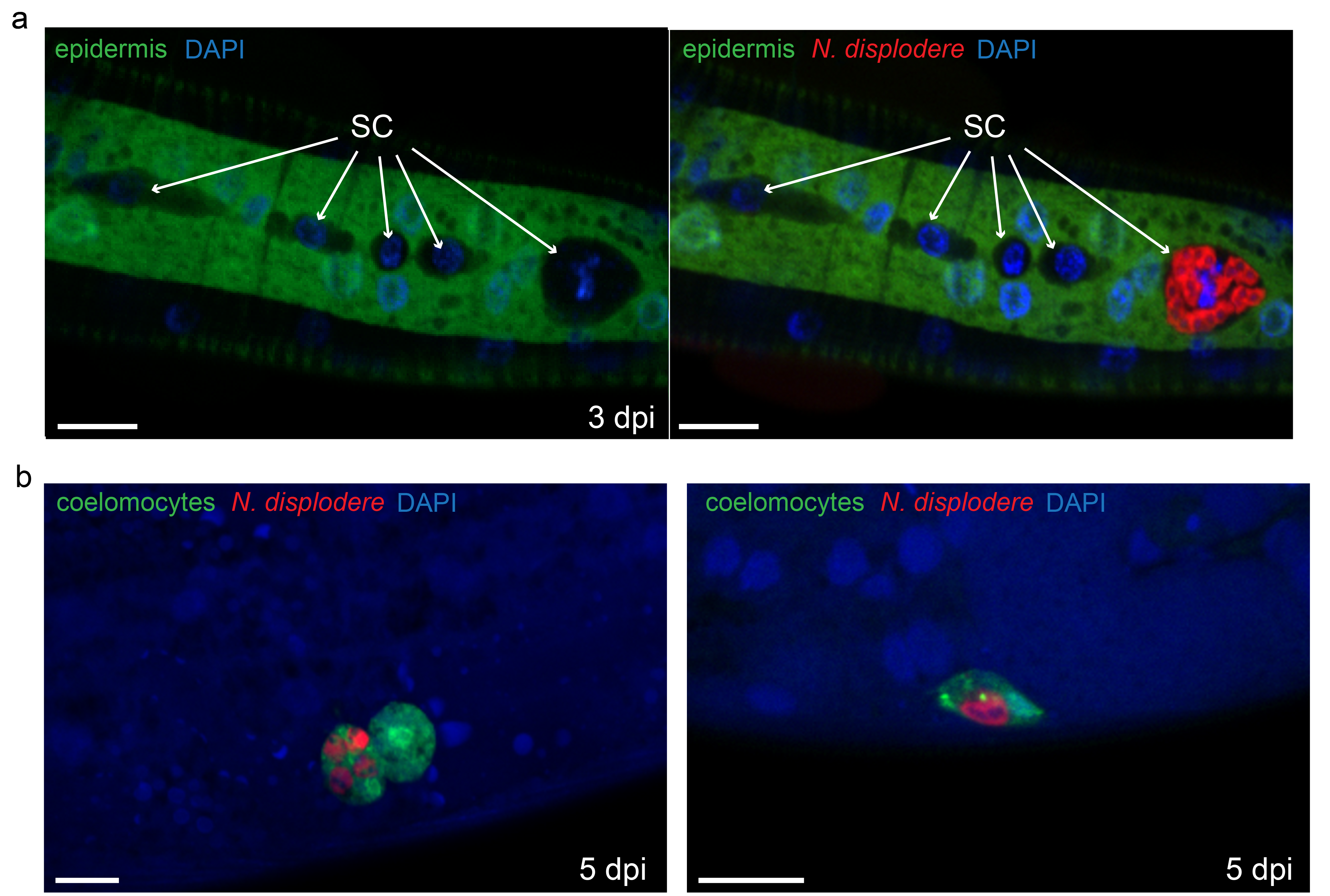

Supplement: S2 Fig — (a) C. elegans strain AU189 expressing GFP in the epidermis was infected with N. displodere and fixed at 3 dpi for N. displodere rRNA FISH and counterstained with DAPI. Seam cells (SC) were identified as nuclei-containing, GFP-negative cells within the GFP-positive epidermis, in the top or bottom plane of the animal laying on its left or right side. Images without (left) or with (right) the red channel (N. displodere FISH) are shown. (b) C. elegans strain OH910 expressing GFP in coelomocytes was infected with N. displodere and fixed at 5 dpi for N. displodere rRNA FISH and counterstained with DAPI. Two images of two separate animals are shown with meronts inside coelomocytes. Scale bars are 10 μm. (TIF) [file ppat.1005724.s002.tif]

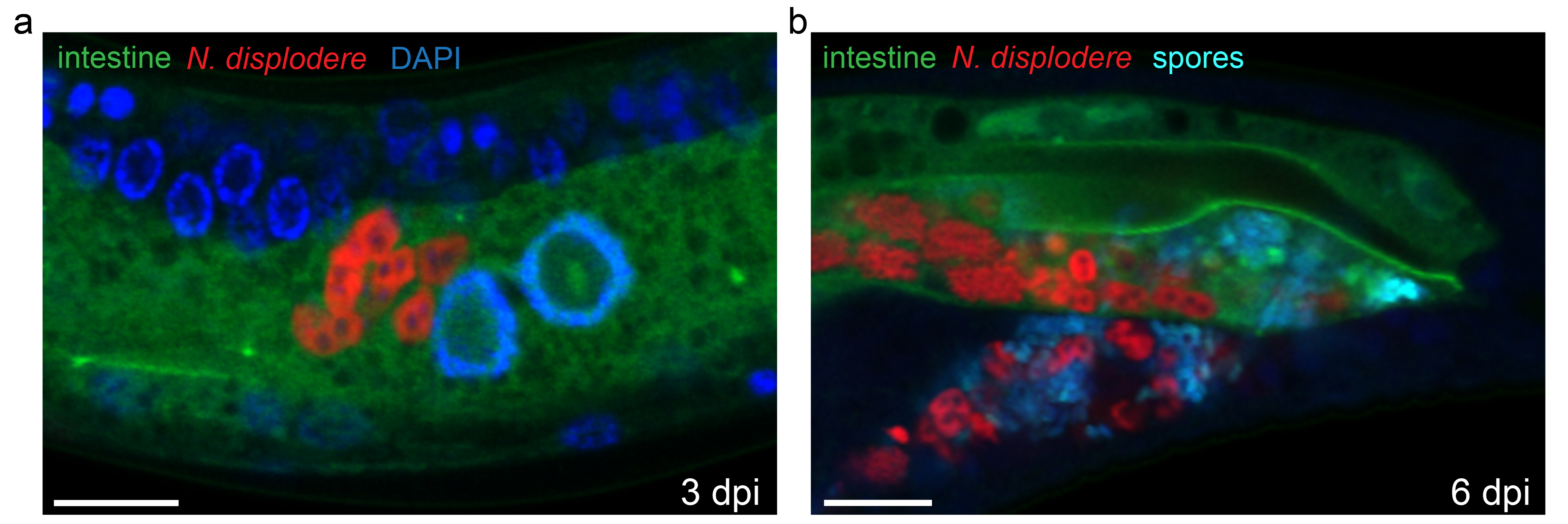

Supplement: S3 Fig — (a) C. elegans intestinal-specific GFP-expression strain ERT413 was infected with N. displodere and fixed at 3 dpi for N. displodere rRNA FISH and DAPI. Meronts are seen in the GFP-labeled intestine. (b) Strain ERT413 was infected with N. displodere and treated as above at 6 dpi, except DY96 was used to stain spores. Meronts and spores are observed both inside and outside of the GFP-labeled intestine. Scale bars are 10 μm. (TIF) [file ppat.1005724.s003.tif]

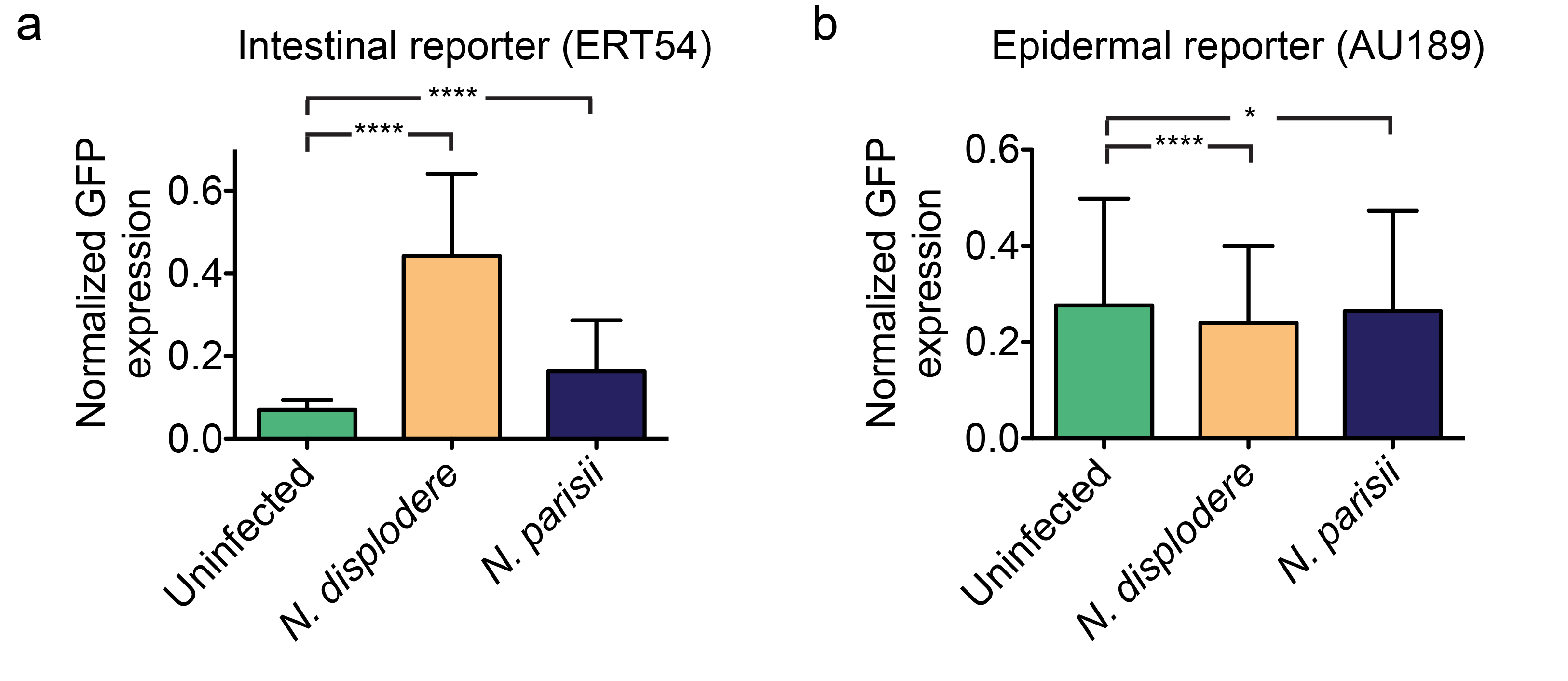

Supplement: S4 Fig — (a) Normalized GFP induction of an intestinal infection reporter strain (ERT54 C17H1.6p::GFP) after N. displodere or N. parisii infection. Signal was normalized by body size using time of flight on the COPAS Biosort. Data are represented as mean values with SD from n = 1800 animals from six replicates across two independent experiments (****p<0.0001, two-tailed Mann-Whitney test). (b) Another independent experiment showing GFP induction of the epidermal damage/infection reporter strain AU189 after N. displodere or N. parisii infection (for the other independent replicates see Fig 5A). Animals were normalized by red fluorescence (pcol12::dsred). Data show mean values with SD from n = 900 animals across three replicates (***p<0.0001, *p = 0.038, two-tailed Mann-Whitney test). (TIF) [file ppat.1005724.s004.tif]

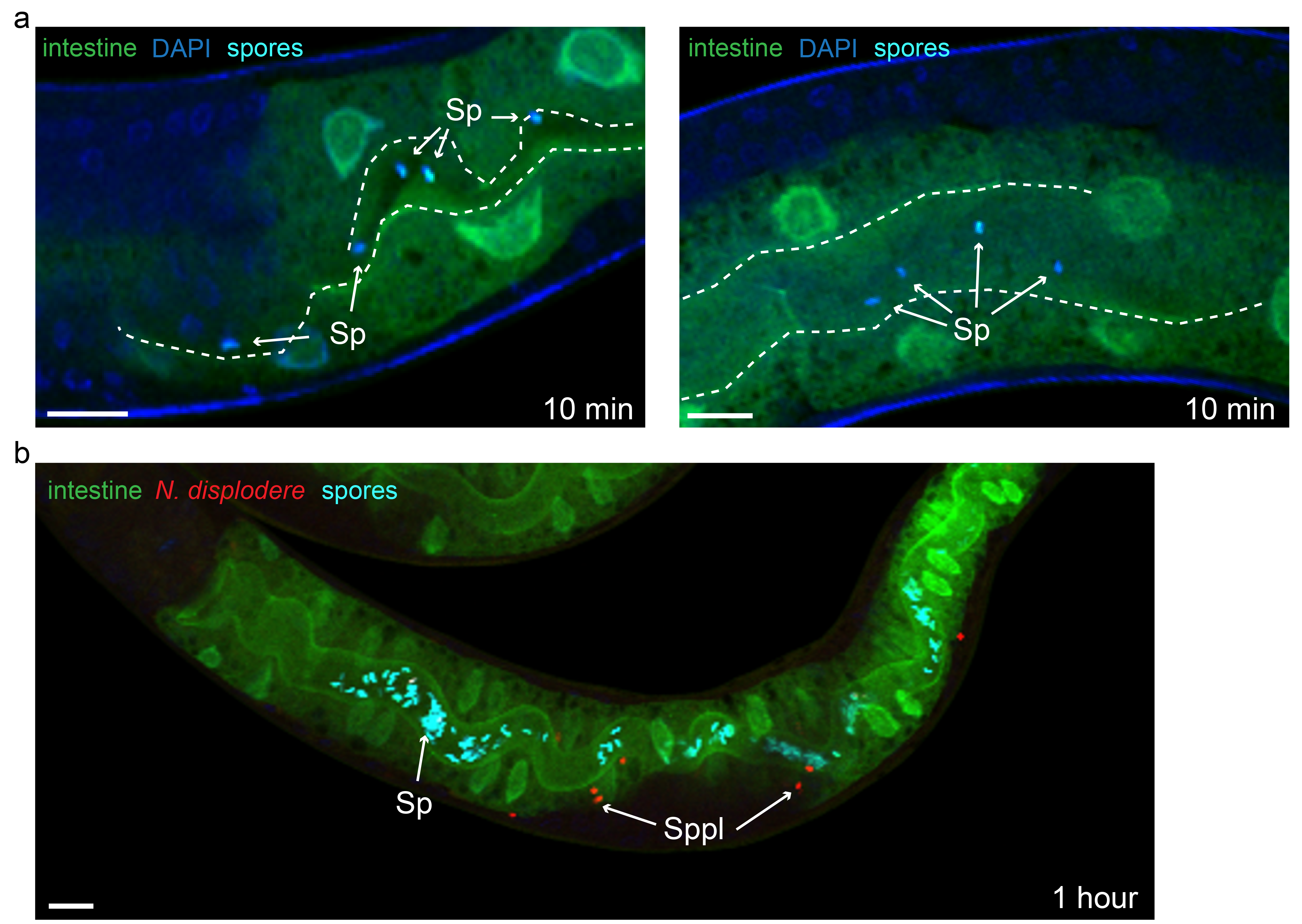

Supplement: S5 Fig — (a) C. elegans strain ERT413 expressing GFP in the intestine was infected at the L4 stage with N. displodere for 10 m and fixed for staining by DAPI (blue) and DY96 (turquoise). Spores (Sp) are seen as DY96-positive oval structures in the intestinal lumen, delineated by dashed lines. (b) C. elegans strain ERT413 expressing GFP in the intestine was infected at the L3 stage with N. displodere for 1 hour and stained for N. displodere rRNA FISH and DY96. Spores are seen only in the intestinal lumen and sporoplasms (Sppl) are indicated. Scale bars are 10 μm. (TIF) [file ppat.1005724.s005.tif]

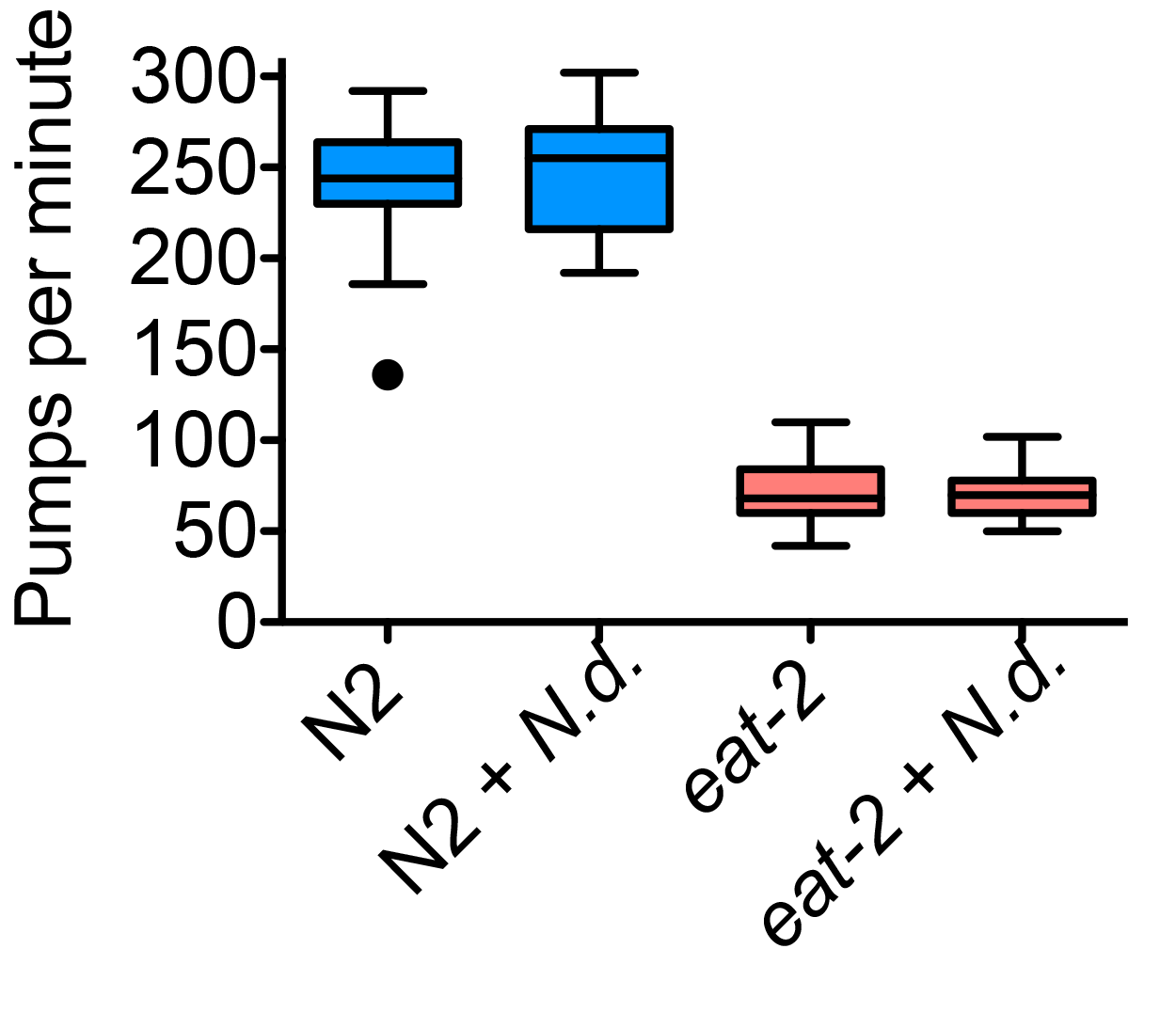

Supplement: S6 Fig — Tukey boxplots of N2 and eat-2 pharyngeal pumping rates with or without N. displodere (N.d.) infection at 15°C from n = 50 animals examined across two independent replicates. The lines indicate the median, the box extends from the 25th to 75th percentiles, and the whiskers extend to the minimum and maximum data point, excluding an outlier (indicated with a black dot). (TIF) [file ppat.1005724.s006.tif]

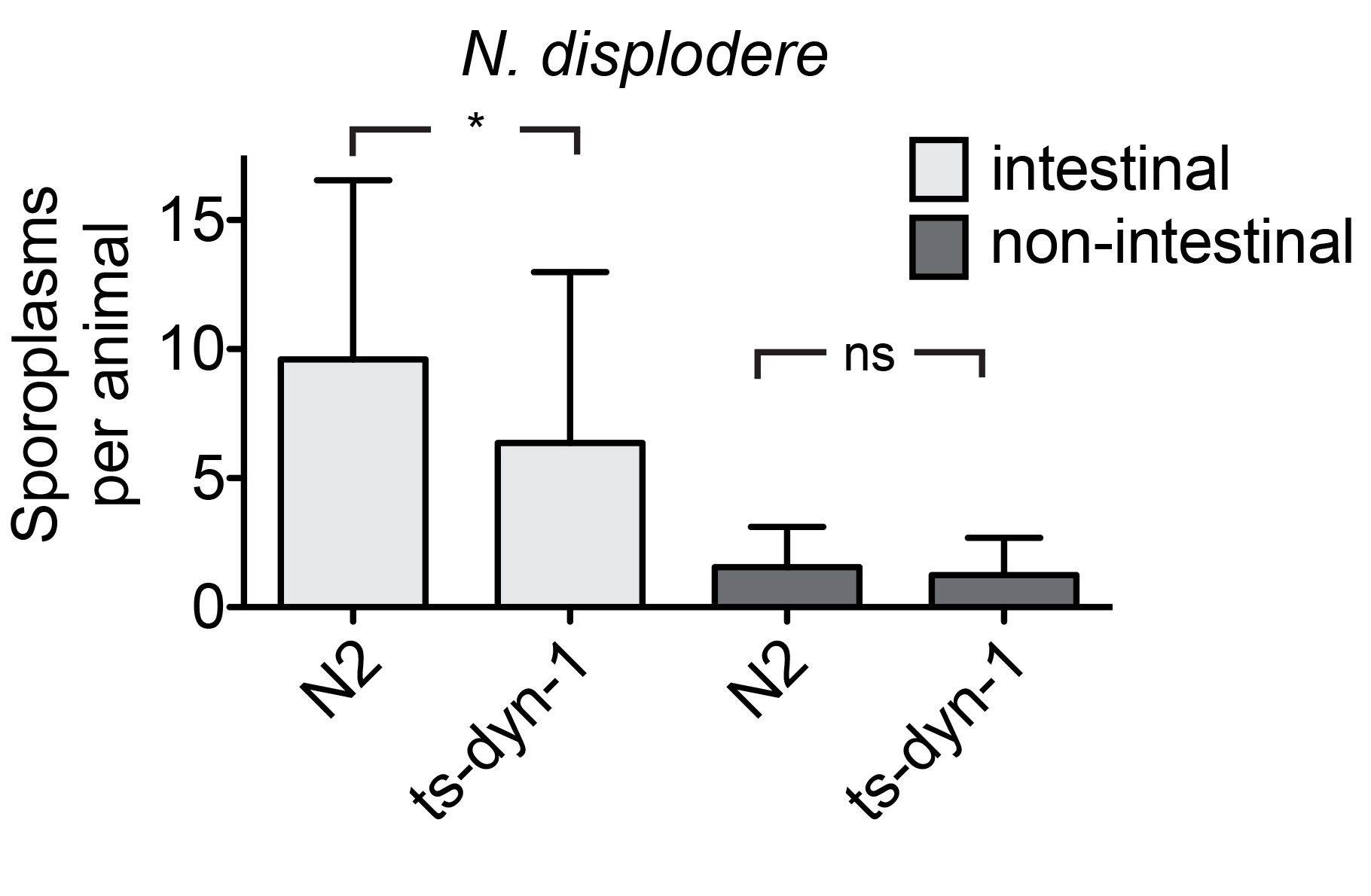

Supplement: S7 Fig — Comparison of the number of invasion events (counted as sporoplasms) occurring in dyn-1(ts) and N2 animals after N. displodere infection at 20°C for 30 min. Events were counted as either intestinal or non-intestinal. Data are represented as mean values with SD from n = 25 animals from one experiment (*p = 0.038, ns = not significant (p = 0.432), two-tailed Mann-Whitney test). (TIF) [file ppat.1005724.s007.tif]

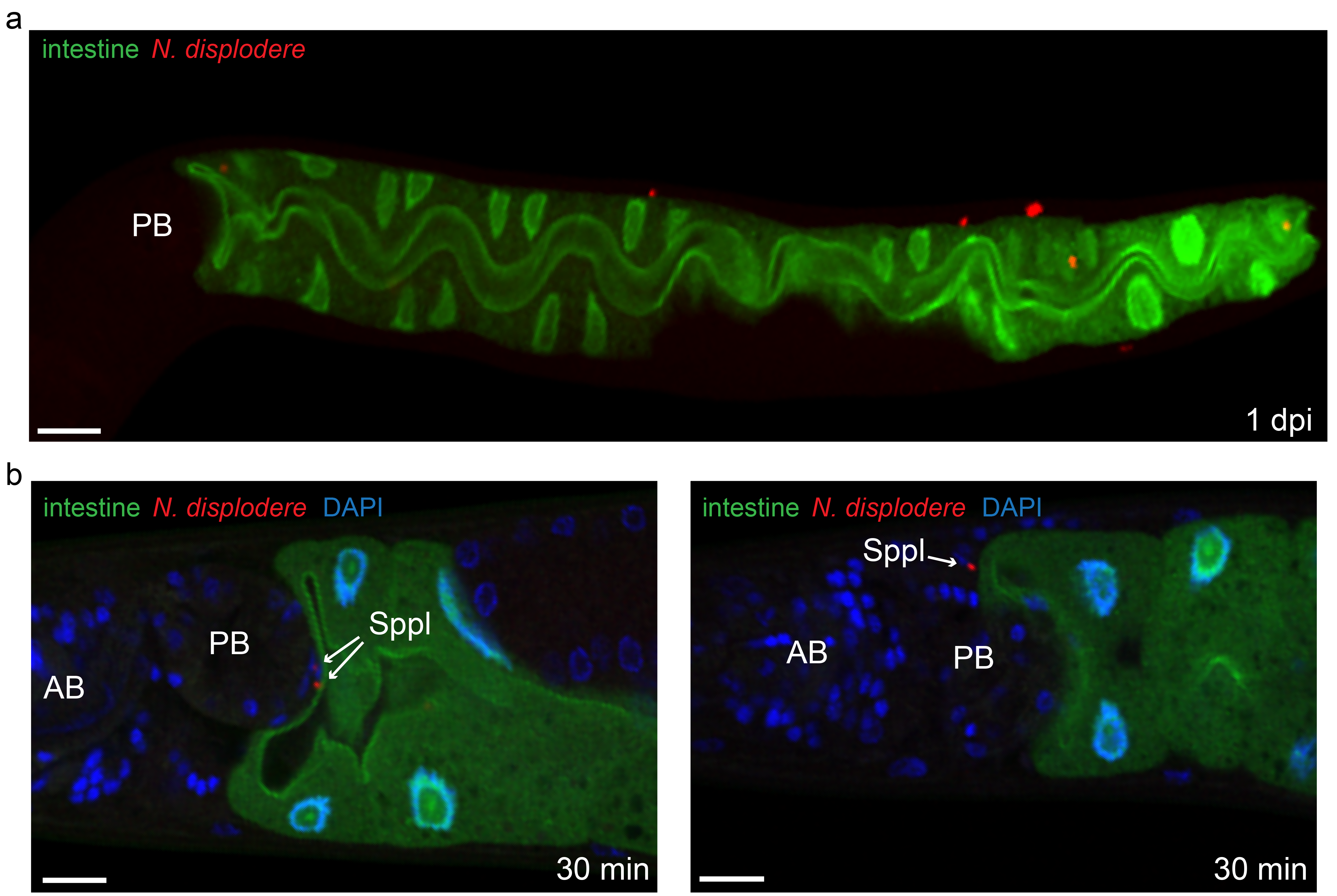

Supplement: S8 Fig — (a) C. elegans strain ERT413 expressing GFP in the intestine was infected as L1 larvae with N. displodere and fixed at 1 dpi for N. displodere rRNA FISH. Sporoplasms are seen inside and outside of the GFP-labeled intestine, in close proximity to the intestine, but never anterior to the posterior bulb (PB). (b) Strain ERT413 was infected as adults for 30 min with N. displodere and treated as above. Sporoplasms (Sppl) in the anterior region are observed in close proximity to the wide intestinal lumen at the anterior part of the intestine. The anterior bulb (AB) and posterior bulb (PB) of the pharynx are indicated. Scale bars are 10 μm. (TIF) [file ppat.1005724.s008.tif]

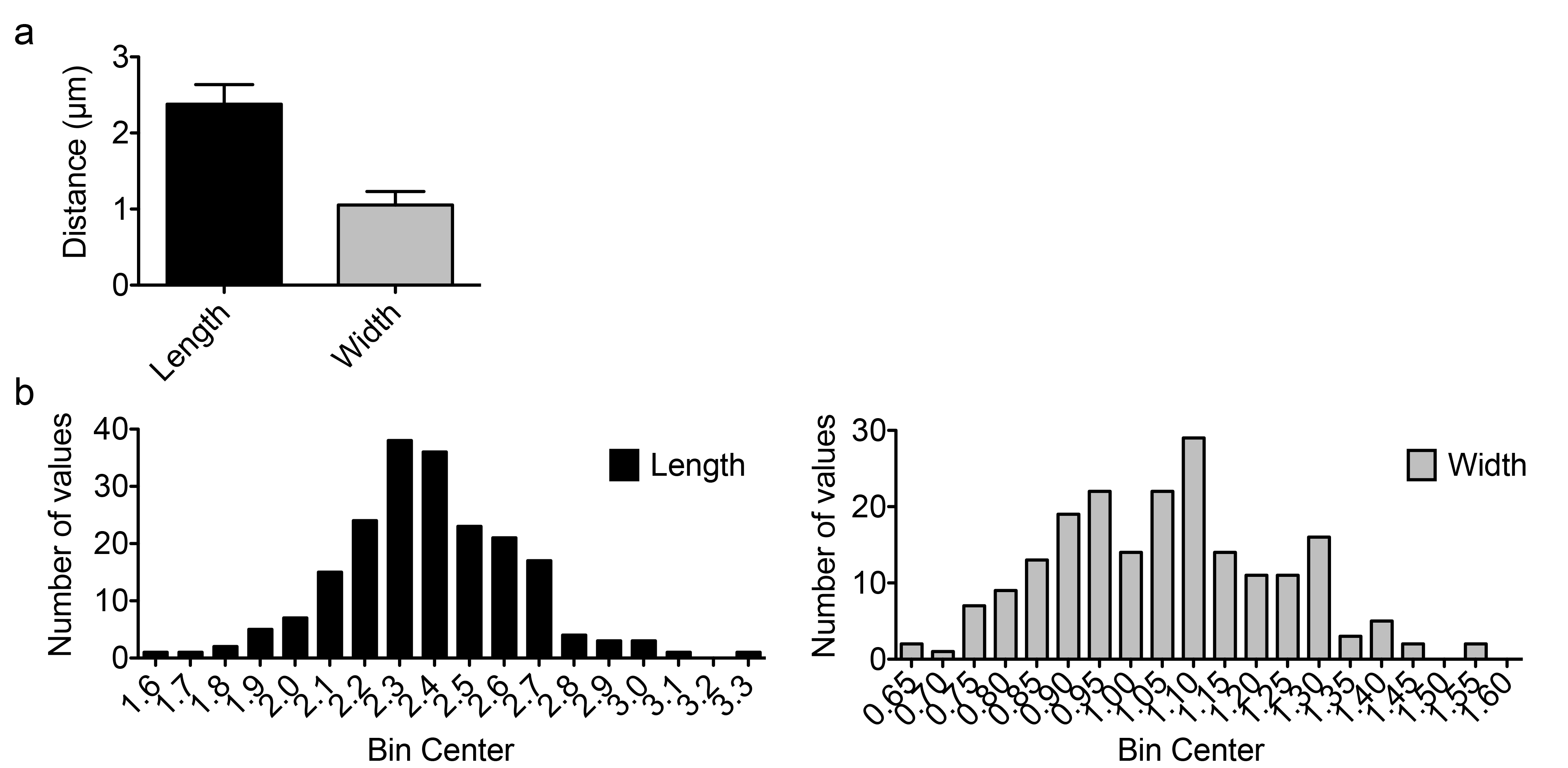

Supplement: S9 Fig — (a) The graph depicts mean length and width of N. displodere spores with SD from n = 202 CFW-stained spores. (b) The histogram depicts the frequency distribution of N. displodere spore lengths (left) and widths (right). (TIF) [file ppat.1005724.s009.tif]

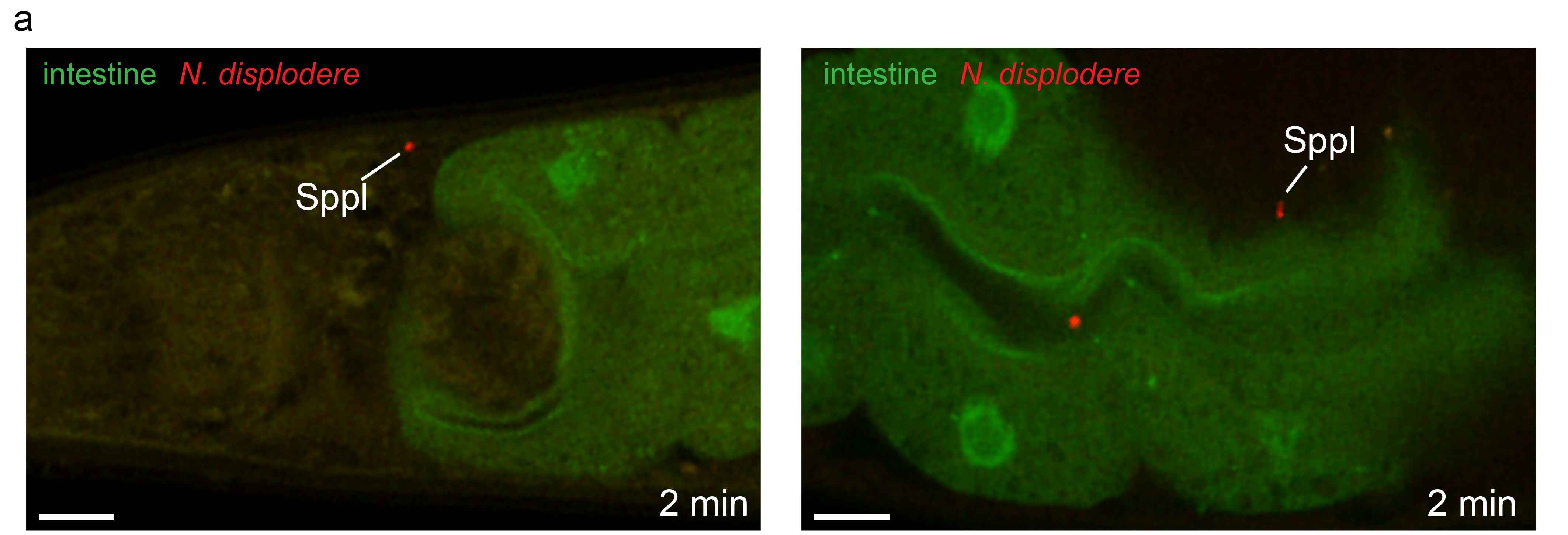

Supplement: S10 Fig — C. elegans strain ERT413 expressing GFP in the intestine was infected as L4 larvae with N. displodere and fixed at 2 minutes post-infection for N. displodere rRNA FISH. Sporoplasms (Sppl) are seen outside of the GFP-labeled intestine. Scale bar = 10 μm (TIF) [file ppat.1005724.s010.tif]

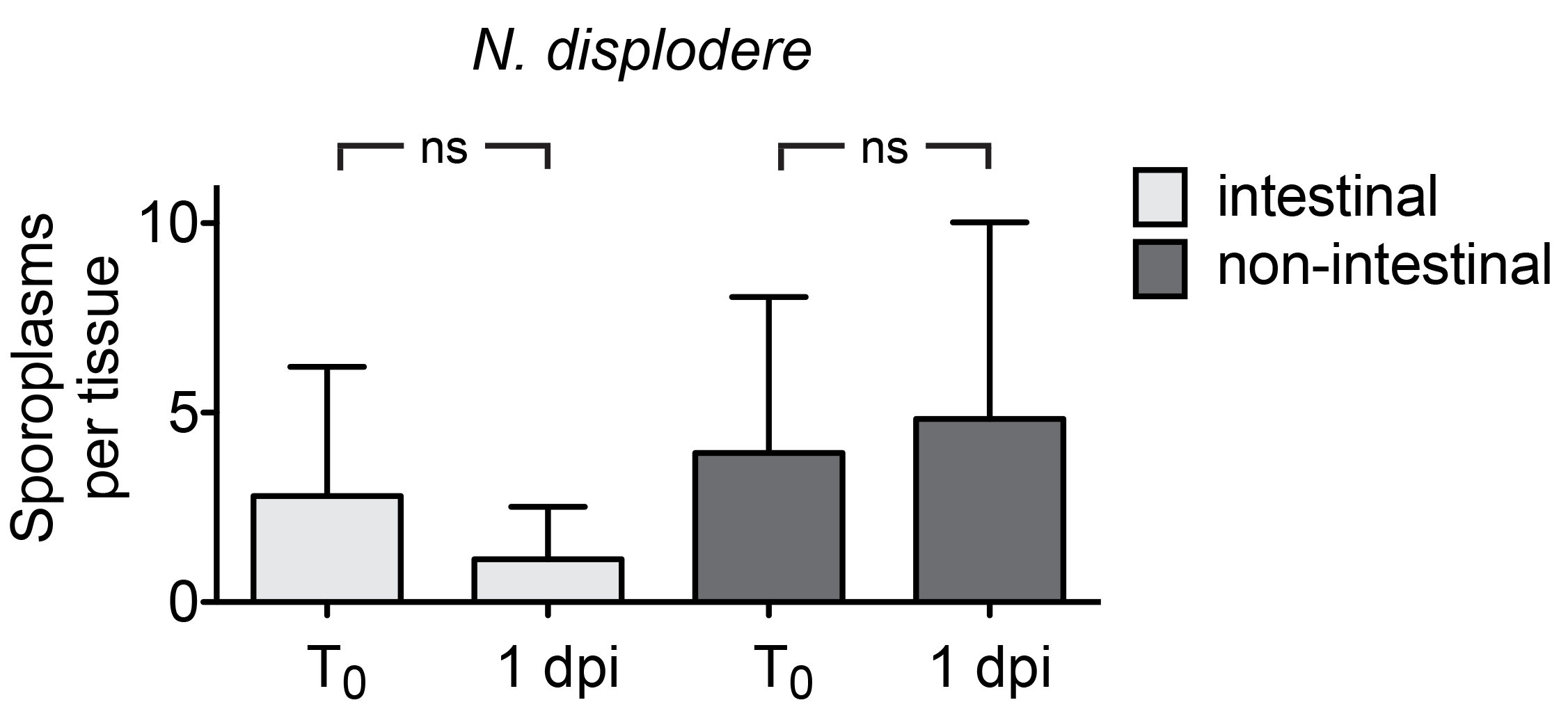

Supplement: S11 Fig — Comparison of the number of invasion events (counted as sporoplasms) occurring when L3 animals of ERT413 were infected with N. displodere spores for 1 hour and immediately fixed (T0) or washed to remove spores and allowed to grow at 15°C for an additional 23 hours (1 dpi). Events were counted as either intestinal or non-intestinal based on localization with intestinal GFP. Data are represented as mean values with SD from n = 30 animals from one experiment (ns = not significant, two-tailed Mann-Whitney test). (TIF) [file ppat.1005724.s011.tif]

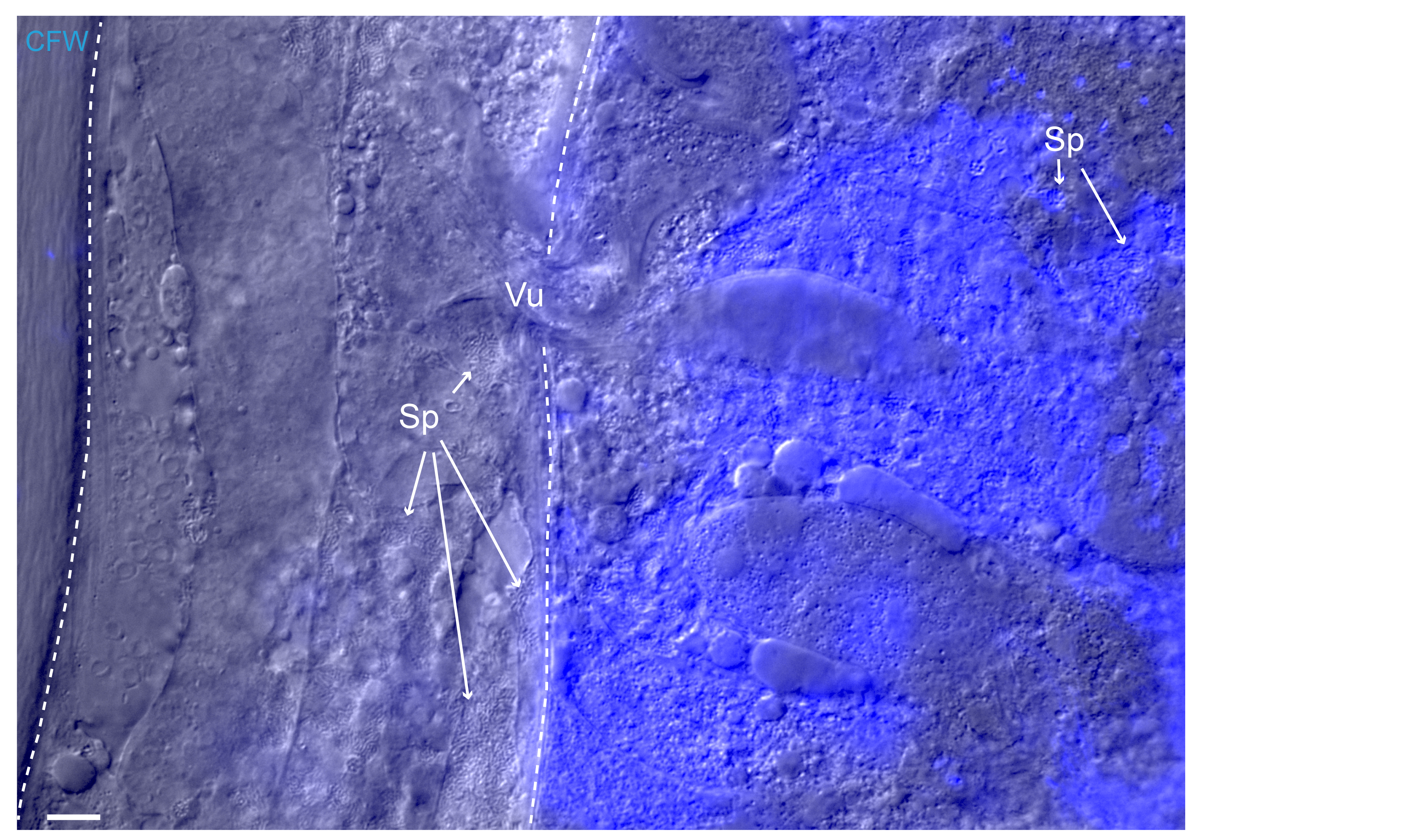

Supplement: S12 Fig — A micrograph of a live animal infected for 9 days with N. displodere and observed to have a burst vulva on the plate. This animal was picked to an agarose pad on a slide, calcofluor white (CFW) at 1:100 dilution was added to stain external spores, and imaged with a 63x objective on a Zeiss AxioImager M1 upright microscope. The vulva (Vu) is seen with C. elegans tissue seen inside the animal (outline with dashed lines) and outside the animal. N. displodere spores (Sp) are seen stained with CFW outside of the animal. (TIF) [file ppat.1005724.s012.tif]

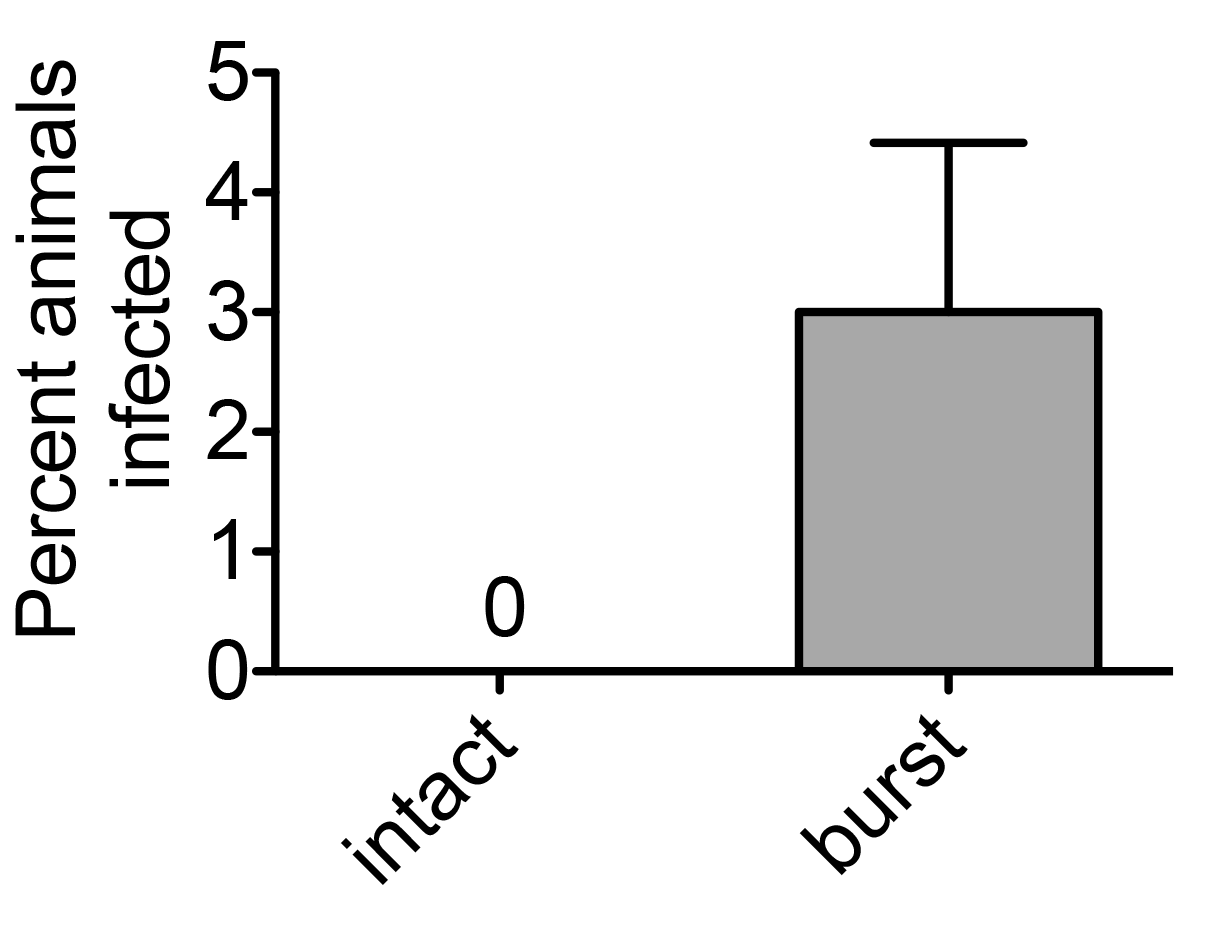

Supplement: S13 Fig — Wild-type N2 animals infected with N. displodere for 8 days were split into two groups, intact animals and animals with a burst vulva. Twenty animals of each group were transferred to a fresh plate with starved ERT413 animals for 4 hours and then removed. ERT413 animals were grown at 15°C for 4 days and fixed for N. displodere rRNA FISH. Fifty GFP-positive animals were inspected for N. displodere infection per replicate. Data are represented as mean values with SD from two replicates from one experiment. (TIF) [file ppat.1005724.s013.tif]

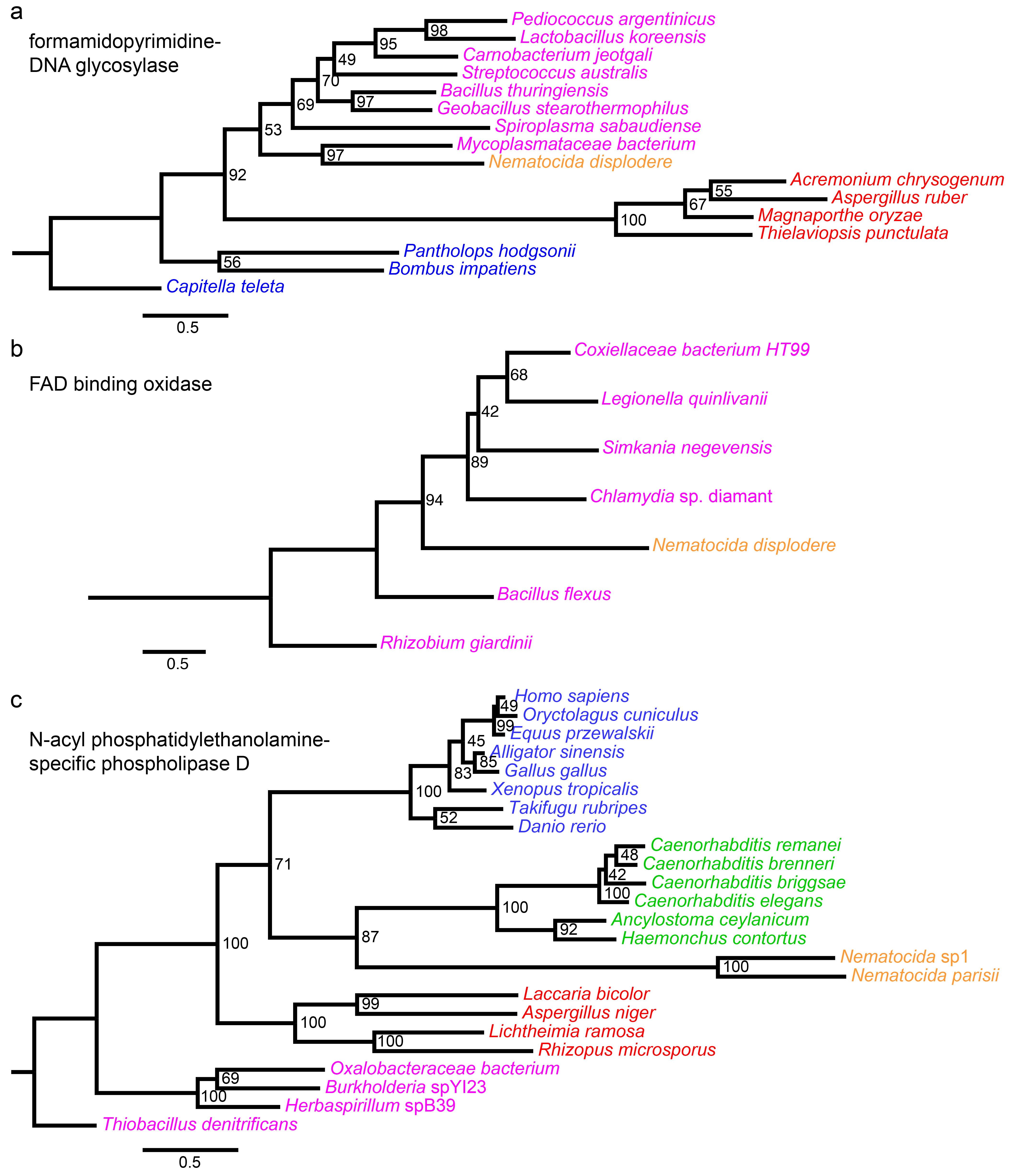

Supplement: S14 Fig — Phylogenetic trees of possible horizontal gene transfer involving (a) formamidopyrimidine-DNA glycosylase (NEDG_02224, N. displodere), (b) FAD binding oxidase (NEDG_00514, N. displodere), and (c) NAPE-2 (NEPG_01645, N. parisii; NERG_00761, N. sp. 1). Representative homologs of each Nematocida enzyme were used to infer trees with RAxML 8.2.4 using the PROTGAMMALG model and 1000 bootstrap replicates. Proteins belonging to the following groups were colored: fungi (red), metazoans (blue), microsporidia (orange), bacteria (magenta), and nematodes (green). Bootstrap supports are indicated next to each node. Scale bars indicate changes per site. The tree was created with FigTree 1.4.2 (http://tree.bio.ed.ac.uk/software/figtree/). (TIF) [file ppat.1005724.s014.tif]
