# Supplementary material for: Discovery of a Natural Microsporidian Pathogen with a Broad Tissue Tropism in Caenorhabditis elegans
Source: PLoS Pathog. 2016 Jun 30;12(6):e1005724. doi: 10.1371/journal.ppat.1005724 (PMC4928854; doi:10.1371/journal.ppat.1005724)
Supplement: S1 File — (DOCX) [file ppat.1005724.s016.docx]

**Supplementary File 1: Taxonomic summary of *N. displodere***

***Nematocida displodere* defines a new species (urn:lsid:zoobank.org:act:35CF055F-C311-4D9B-BFF0-B7B09FC441E4).** Its host is the nematode, *Caenorhabditis elegans*. It is orally transmitted, with no evidence of vertical transmission. There was no continuous route detected for new spore exit from infected nematodes. One means by which spores exit the host to be transmitted to new hosts is through infection-induced vulva bursting.

**Life cycle and symptoms in the host.** Symptoms of infection are detected by Nomarski light microscopy as large roundish vesicle structures with multiple circular nuclei often in association with oblong spores. Symptoms are most often seen in tissues of the body wall of *C. elegans,* anywhere along the anterior/posterior axis, although the first occurrences of symptoms are seen along this axis between the *C. elegans* posterior bulb and anus. Occasionally these symptoms are seen in the intestine, but usually at late stages of infection*.* The replicative stages (meronts) are seen by Nomarski as early as 3 dpi at 15°C, and their association with spores are seen as early as 5 dpi at 15°C. Spores are usually seen in groups, and often appear to be in membrane-enclosed structures, as Brownian motion of groups of spores in live, infected animals is limited to small circular-like areas.

There were multiple sites of infection observed by rRNA FISH, including epidermis, muscle, neurons, intestine, coelomocytes, and seam cells. Newly formed spores were seen predominantly in the muscle and epidermis, but were occasionally seen in the neurons and intestine. All post-embryonic stages showed signs of infection, except the non-feeding dauer stage.

Only one spore size was observed, measuring 2.38 µm (+/- 0.26 µm) long and 1.03 µm (+/- 0.18 µm) wide. Externalized polar tubes were measured as 12.55 µm (+/- 3.20 µm), while internal polar tubes were observed by TEM with up to five polar tube coils per spores seen in radial cross-sections.

The type strain was isolated from a rotting *Asteraceae* stem near the Viosne stream in Santeuil, Val-d’Oise, Île-de-France, France (GPS coordinates: 49.12165, 1.95101) on September 30, 2014.

The etymology of the type species name *N. displodere* is based on the host infection phenotype whereby infected nematodes burst from the vulva and release spores.
